# The effect of the anticholinergic burden on duration and severity of delirium in older hip‐surgery patients with and without haloperidol prophylaxis: A post hoc analysis

**DOI:** 10.1002/brb3.2404

**Published:** 2021-11-10

**Authors:** Monique P. H. Tillemans, Madelon H. Butterhoff‐Terlingen, Rutger Stuffken, Ralph Vreeswijk, Toine C. G. Egberts, Kees J. Kalisvaart

**Affiliations:** ^1^ Stichting Apotheek der Haarlemse ziekenhuizen Haarlem The Netherlands; ^2^ Department of Clinical Pharmacy Ter Gooi Ziekenhuizen Hilversum The Netherlands; ^3^ Department of Geriatric Medicine Spaarne Gasthuis Haarlem The Netherlands; ^4^ Department of Clinical Pharmacy University Medical Center Utrecht Utrecht The Netherlands

**Keywords:** anticholinergic burden, delirium, haloperidol, prophylaxis

## Abstract

**Background:**

Anticholinergic acting drugs have been associated with delirium in older patients.

**Objective:**

To examine the association between the anticholinergic burden (ACB) and the duration and severity of delirium in older hip‐surgery patients with or without haloperidol prophylaxis.

**Methods:**

Older patients with a postoperative delirium following hip surgery from a randomized controlled trial investigating the effects of haloperidol prophylaxis on delirium incidence were included in this study. The ACB was quantified using two different tools, the Anticholinergic Drug Scale and an Expert Panel. Using linear regression, the association between the ACB and delirium was analyzed.

**Results:**

Overall delirium duration and severity were not significantly associated with the ACB. Also, no statistically significant differences were found in delirium duration or severity between the placebo and haloperidol treatment groups for the ACB groups. The protective effect of haloperidol on delirium duration and severity however tended to be present in patients with no or a low ACB but not or to a lesser extent in patients with an intermediate to high ACB.

**Conclusions:**

The ACB was not significantly associated with delirium duration or severity. Haloperidol prophylaxis tended to shorten delirium duration and decrease delirium severity in patients with no or a low ACB. To further explore the influence of anticholinergic acting drugs on delirium duration and severity and the effect of concomitant haloperidol use, additional research with a higher haloperidol dose, a larger study population, and ACB quantification taking drug exposure into account is warranted.

## Abstract 2

In this study we explored the effect of the anticholinergic burden in older hip‐surgery patients on delirium duration and severity with and without the use of haloperidol prophylaxis to gain understanding of the association between anticholinergic burden, delirium, and haloperidol prophylaxis.

## 1 INTRODUCTION

It is estimated that between 3% and 29% of the patients will develop a delirium during hospitalization (Siddiqi et al., 2006). One of the theories on the pathophysiology of delirium is a change in the neurotransmitter systems, resulting in a deficit of acetylcholine and an excess of dopamine. In addition, some patients tend to be more susceptible to delirium in case of a stressor event such as surgery than other patients. Predisposing factors for delirium are cognitive impairment, old age, severe illness, and use of certain drugs. In approximately 12%–39% of the patients the use of particular drugs is thought to be the cause of the delirium (Alagiakrishnan & Wiens, 2004; van Meenen et al., 2014; Young & Inouye, 2007).

Anticholinergic acting drugs are one of the drug classes that have been associated with delirium. Of all the drugs used in clinical practice about 100 drugs are known to have clinical relevant anticholinergic properties (Durán et al., 2013; Tune, 2001). The extent of the anticholinergic effects depend primarily on the drug's affinity for the muscarinic receptor. Muscarinic receptors are located all over the human body and depending on the location and receptor subtype (M1–M5) different physiological responses are mediated. In the brain, the M1 receptor takes part in higher cognitive functions such as memory and learning. As cholinergic transmission enhances cognitive functions, central anticholinergic effects display opposite effects like memory impairment and confusion. Especially older patients are more susceptible to anticholinergic effects as, with increasing age, the number of cholinergic neurons and receptors declines, the blood–brain barrier becomes more permeable, and hepatic and renal clearance decreases resulting in higher blood levels and prolonged exposure (Abrams et al., 2006; Chew et al., 2008; Nishtala et al., 2016; Schliebs & Arendt, 2011).

The risk of anticholinergic adverse effects also increases when several anticholinergic acting drugs are used concomitantly. The association between the anticholinergic burden (ACB) and delirium however remains unclear as some studies have found a high ACB to be a risk factor for delirium, whereas others have not (Best et al., 2013; Fox et al., 2014; Han et al., 2001; Mangoni et al., 2013; Naja et al., 2016). In a previous study we assessed the association of the ACB on delirium, but an association was not found (Butterhoff‐Terlingen et al., 2009). Whether the ACB has an effect on the duration or severity of the delirium once it has occurred has, to the best of our knowledge, never been studied. It has been hypothesized that the use of anticholinergic acting drugs could increase the imbalance between acetylcholine and dopamine in the brain and therefore might sustain or aggravate the delirium. Treatment of the delirium with haloperidol on the other hand may reduce delirium duration or severity by correcting the imbalance between acetylcholine and dopamine. The objective of this study is to explore the association between the ACB on the duration and severity of delirium in older hip‐surgery patients and to assess the effect of haloperidol prophylaxis.

## 2 MATERIALS AND METHODS

### 2.1 Setting and study design

Data for these post hoc analysis were derived from a randomized, double blind, placebo controlled trial investigating the effects of haloperidol prophylaxis on delirium in elderly patients undergoing hip surgery by Kalisvaart et al. Details on the design and results of this study have been described elsewhere (Kalisvaart et al., 2005). In short, eligible patients were 70 years of age or over with an intermediate or high risk for postoperative delirium and admitted for acute or elective hip surgery. The original study was carried out between August 2000 and August 2002 at a 915‐bed teaching hospital in the Netherlands and was approved by the regional research Ethics Committee (METC Noord‐Holland). Risk classification for postoperative delirium was determined by the presence of four risk factors: visual impairment, severity of illness (APACHE II score), cognitive impairment (Mini Mental State Examination [MMSE]), and dehydration. Upon admission after informed consent had been obtained, patients were randomly assigned to either haloperidol prophylaxis 0.5 mg three times a day or placebo until 3 days after surgery. On admission, the trial medication was started and continued until 3 days after surgery. Both the research team and the participants were blinded to the treatment group and blinding was maintained during the study. The level of adherence to the allocated study medication was recorded daily by the clinical staff. Patients were assessed daily using the MMSE, Delirium Rating Scale (DRS‐R‐98), and the Digit Span test to enable delirium diagnosis by DSM‐IV and Confusion Assessment Method (CAM) criteria (Inouye et al., 1990; Trzepacz et al., 2001). If postoperative delirium occurred haloperidol treatment was continued according to standard procedures and delirium duration and severity assessments were executed daily.

### 2.2 Study participants

All patients who developed a postoperative delirium within 3 days after surgery in the original study were included in the current study.

### 2.3 Assessment of anticholinergic burden

Upon hospital admission the patient's medication use was registered. These data were used to quantify the ACB. There are at least 12 different screening tools and 1 equation known to quantify the ACB with considerable variation among these tools (Carnahan et al., 2006; Kashyap et al., 2014; Mayer et al., 2015; Rudolph et al., 2008; Villalba‐Moreno et al., 2016). Some of these screening tools are based on serum anticholinergic activity (SAA) others on the drugs' anticholinergic properties and professional consensus. In most tools, drugs are ranked in an ordinal fashion from “0” indicating no known anticholinergic activity to “3” indicating considerable anticholinergic effects. With the ACB being the sum of the individual drug scores a patient concomitantly uses. In this study two different tools to quantify the ACB were used. The first screening tool was the Anticholinergic Drug Scale (ADS). Originally, the ADS was known as the Clinician‐rated ACH score and comprised 340 drugs that were rated in an ordinal fashion 0–3 based on literature information and expert panel consensus. By summing the awarded ADS drug scores, the ACB was quantified in 297 patients. Concomitantly the SAA was measured in these patients, showing an association between the patient's ACB and anticholinergic serum activity (Kersten et al., 2012). The second tool was a Dutch expert panel consisting of two geriatricians, three hospital pharmacists, and a hospital pharmacist in training. Comparable to the ADS, the patient's medication was reviewed by the expert panel. Each drug was awarded an anticholinergic score between 0 and 2 (no, weak, and strong anticholinergic properties) based on known anticholinergic side effects and clinical experience consensus on the anticholinergic score was reached by the expert panel. The sum of the individual scores yielded the ACB.

### 2.4 Outcome measures

#### 2.4.1 Delirium duration

Delirium duration was measured in days. With day 1 being the first day the delirium was diagnosed and the last day, the last day that the patient still met the criteria for delirium.

#### 2.4.2 Delirium severity

To assess delirium severity the DRS was used. The DRS comprises 13 items to assess delirium severity and 3 diagnostic items and has been validated in clinical practice. It ranges from 0, no severity, to 45, very high severity. The maximum delirium severity was defined as the highest DRS score measured per patient.

#### 2.4.3 Statistical analyses

Delirium duration and severity were analyzed using linear regression. A two‐tailed *p*‐value < .05 was considered to indicate statistical significance. Data was verified for normal distribution using the Kolmogorov–Smirnov test. The median delirium duration and severity were determined per treatment group. Statistical analysis was performed using SPSS for Windows, version 21 (SPSS, Inc., Chicago, IL).

## 3 RESULTS

In the original study medication use was registered upon hospital admission in 397 of the 430 patients. Of these 397 patients, 68 (15.8%) developed an acute postoperative delirium within 3 days after surgery and were included in this study. These patients used a total of 92 different medicines, with a mean of 3.7 medicines per patient. There were no significant differences between the patients who received haloperidol or placebo in baseline characteristics (Table 1). Independent of the method used to quantify the ACB, 75% of the patients had an ACB score ≤ 1 and about 10% an ACB score ≥ 3 (Table 2). Two patients had postoperative delirium, but treatment randomization was unmasked due to an emergency. These patients were excluded from the final analyses (*n* = 66). Because of the small number of patients in the intermediate and high ACB groups (groups 2 and ≥3) the ACB was categorized into three groups; 0 = no ACB, 1 = low ACB, and ≥2 intermediate/high ACB for the final analyses.

**TABLE 1:** Characteristics of the patients with postoperative delirium (*n* = 68)

	Placebo	Haloperidol	
Characteristics	(*n* = 37)	(*n* = 31)	*p‐*value
Age, mean	82.3	82.6	.86
Female, *n* (%)	24 (64.9)	23 (74.2)	.41
Acute surgery, *n* (%)	19 (54.8)	17 (54.8)	.77
Mini Mental State Examination, mean	20.6	21.7	.30
APACHE II score, mean	14.9	15.4	.56
Visual acuity, mean	0.36	0.31	.11
Blood urea nitrogen/creatinine ratio, mean	11.7	12.2	.60

**TABLE 2:** Anticholinergic burden quantified by ADS and Expert Panel (*n* = 68)

ACB score	ADS placebo	ADS haloperidol	Total (%)	Expert Panel placebo	Expert Panel haloperidol	Total (%)
0	18	17	35 (51.5)	13	13	26 (38.2)
1	9	7	16 (23.5)	15	10	25 (36.8)
2	8	2	10 (14.7)	7	2	9 (13.2)
≥3	2	5	7 (10.3)	2	6	8 (11.8)

*Abbreviations*: ACB, anticholinergic burden; ADS, Anticholinergic Drug Scale.

**FIGURE 1:**
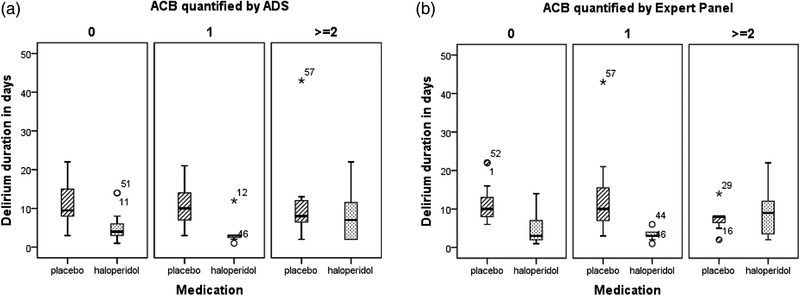
Distribution of delirium duration for the three ACB groups for the placebo and haloperidol treatment groups. The ACB quantified by (a) ADS and (b) Expert Panel. The treatment allocation, placebo, or haloperidol, is depicted on the *x*‐axis and delirium duration in days on the *y*‐axis. The columns represent the three ACB groups; 0 = no ACB, 1 = low ACB, and ≥2 intermediate/high ACB

Overall delirium duration and severity were not significantly associated with the ACB independent of the method used to quantify the ACB. Also, no statistically significant differences were found in delirium duration and or severity between the placebo and haloperidol treatment groups for the ACB groups. The distribution of delirium duration per treatment group divided per ACB is presented in Figure 1. The protective effect of haloperidol on delirium duration found in the original study by Kalisvaart et al. tended to be present for patients with an ACB of 0 and 1 but not for patients with an intermediate to high ACB ≥ 2. The median delirium duration for patients with an ACB of 0 or 1 quantified by ADS was 9.5 and 10 days, respectively, for placebo against 4 and 3 days for haloperidol treated patients. In the intermediate to high ACB group the median delirium duration was 8 days for placebo and 8 days for haloperidol treated patients. Quantification of the ACB by Expert Panel showed similar results. The median delirium duration for patients with an ACB of 0 or 1 was 10 days for placebo and 3 days for haloperidol in both ACB groups versus 8 and 9 days for patients with an intermediate to high ACB for placebo and haloperidol, respectively.

**FIGURE 2:**
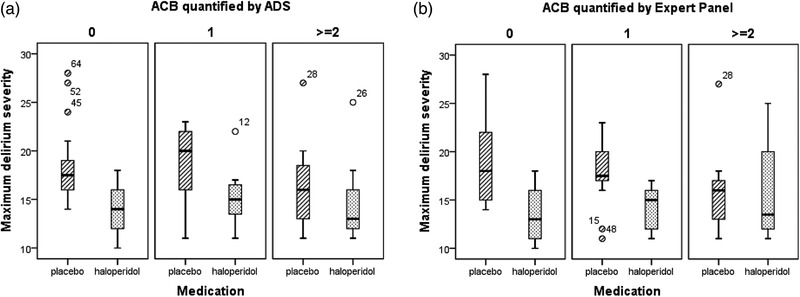
Distribution of the maximum delirium severity for the three ACB groups, for both the placebo and haloperidol treatment groups. The ACB quantified by (a) ADS and (b) Expert Panel. The treatment allocation, placebo, or haloperidol, is depicted on the *x*‐axis and maximum delirium severity on the *y*‐axis. The columns represent the three ACB groups; 0 = no ACB, 1 = low ACB, and ≥2 intermediate/high ACB

The distribution of the maximum delirium severity per treatment group and divided per ACB is presented in Figure 2. The protective effect of haloperidol on delirium severity demonstrated in the original study also tended to be less in patients with an intermediate to high ACB. The median of the maximum delirium severity was 17.5 and 20 for patients with an ACB of 0 and 1 quantified by ADS in the placebo treatment group and 14 respectively 15 for the haloperidol treatment group. For the intermediate to high ACB, the median of the maximum delirium severity was 16.5 for the placebo treated patients versus 13 in the haloperidol treated patients. Similar results were obtained when the ACB was quantified by the Expert Panel. The median of the maximum delirium severity was 17.5 and 18 for placebo treated patients with an ACB of 0 and 1 compared to 13 and 15 for the haloperidol treated patients. For patients with an intermediate to high ACB, the maximum delirium severity was 16 for the placebo treated patients versus 13 in the haloperidol treated patients.

## 4 DISCUSSION

To the best of our knowledge this is the first study to explore the association between the ACB and delirium duration and severity with and without haloperidol prophylaxis. Overall, the ACB was not significantly associated with delirium duration or delirium severity. The protective effect of haloperidol on delirium duration and severity however tended to be present in patients with “no” or a “low” ACB but not or to a lesser extent in patients with an “intermediate” to “high” ACB.

One of the strengths of this study is the inclusion of older patients at risk for delirium and susceptible to the adverse effects of anticholinergic acting drugs. Also, data from a randomized controlled trial were used and two different tools were used to quantify the ACB. In the original study a significant association between haloperidol and delirium duration and severity was found, but not on delirium incidence. It was hypothesized that the haloperidol dose of 0.5 mg t.i.d. was too low for the primary prevention of acute postoperative delirium. The low haloperidol dose used in the original study might also explain the trends found in this study. The haloperidol appears to have a protective effect on delirium duration and severity in patients with no or a low ACB, but less in patients with an intermediate to high ACB. The haloperidol dose of 0.5 mg t.i.d. might not have been enough to correct the neurotransmitter imbalance between acetylcholine and dopamine in patients with an intermediate to high ACB.

There are however some limitations that need to be considered. One limitation is the small sample size. Only 66 patients of the original study could be included in this post hoc analysis. These data however were extracted from a randomized controlled trial and despite the small sample size a trend was observed. Also, the use of anticholinergic drugs with strong anticholinergic properties was relatively low, as only 10% of the patients had an ACB score ≥ 3. Other studies using the ADS to quantify the ACB reported about 20% of the patients having an ACB score of ≥3. The overall anticholinergic medication use of approximately 40%–50% in this study however was comparable to other studies (Mate et al., 2015).

Another limitation might be the registration of the patients' medication at hospital admission only, as changes in medication during hospitalization are likely (Dauphinot et al., 2014). Most patients, however, had surgery within 24 h after hospital admission and delirium developed within the first 3 days of hospital admission.

Medication use of the included subjects seemed rather low when considering the mean age of 82 years of the included patients. Based on data from the Netherlands institute for Health Services Research (Nivel), 51% of the patients between 75 and 85 years of age use at least 5 different drugs and about 17% uses 10 drugs or more (van Dijk et al., 2009). In this study, only 36.8% of the patients used at least 5 and 7.4% at least 10 different drugs. Also, the mean number of medicines used by these patients was only 3.7. Medication use was however not verified with prescription data from the patient's pharmacy or general practitioner. This may have resulted in underreporting or errors in some of the patient's medication use. Underreporting of medication use might have altered the ACB before and during the delirium. It is however questionable whether individual changes in the ACB would have influenced the trends shown in this study.

In the literature there are at least a dozen different tools described to quantify the ACB. None of these is considered the method of choice as there are quite a few limitations concerning these tools. One of the major limitations of most tools is that drug exposure, for example, administration route, dose, and anticholinergic metabolites are not taken into account when awarding a certain score. Also, all tools assume that the anticholinergic drug scores are additive in a linear fashion, this however is unlikely due to the limited number of muscarinic receptors. Additionally, these tools have been developed in different countries yielding considerable differences in drug selection and grading. Furthermore, all tools using an expert panel review are subject to interpretation bias. The Drug Burden Index (DBI) is the only tool that takes the drug dose into account and therefore seems the most appropriate tool for ACB quantification (Cardwell et al., 2015). The DBI however could not be used in this study as data on the drug doses were not available. Because of the considerable differences in drug selection and grading between the different tools described in the literature, the ACB was quantified using two different tools (Lozano‐Ortega et al., 2020). Besides the ADS, an Expert Panel quantified the ACB as an ACB assessment tool has not been developed in the Netherlands and the existing tools do not contain all the drugs used in the Netherlands. Both the ADS and the Expert Panel identified 51 patients in the “no” or “low” ACB and 17 patients with an “intermediate” to “high” ACB score. As expected, there were differences between these tools in the quantification of the ACB. However, in 64.7% (44 out of 68 patients) both the ADS and the Expert Panel yielded the same ACB score. In addition, the same trends on delirium duration and severity were shown independent of the tool used to quantify the ACB.

## 5 CONCLUSIONS

This is the first study to investigate the effect of the ACB and the influence of haloperidol on delirium duration and severity. A significant association of the ACB on delirium duration or severity, with and without haloperidol treatment, was not found. The use of haloperidol however tended to shorten delirium duration and decrease delirium severity in patients with no or a low ACB. To further explore the influence of anticholinergic acting drugs on delirium duration and severity and the effect of concomitant haloperidol use additional research with a higher haloperidol dose, a larger study population, and ACB quantification taking drug exposure into account is warranted.

## AUTHOR CONTRIBUTIONS

Monique P. H. Tillemans, Kees J. Kalisvaart, and Toine C. G. Egberts were involved in designing the study. Madelon H. Butterhof, Rutger Stuffken, and Ralph Vreeswijk acquired the data. Monique P. H. Tillemans performed the data analysis and wrote the first draft of the manuscript. All authors interpreted the data, critically revised the manuscript, read, and approved the manuscript.

## CONFLICT OF INTEREST

The authors declare that they have no conflict of interest.

### PEER REVIEW

The peer review history for this article is available at https://publons.com/publon/10.1002/brb3.2404

## Data Availability

The data that support the findings of this study are available from the corresponding author upon reasonable request.
